# Gray Matter Volumes in Patients with Chronic Fatigue Syndrome

**DOI:** 10.1155/2015/380615

**Published:** 2015-02-22

**Authors:** Le-wei Tang, Hui Zheng, Liang Chen, Si-yuan Zhou, Wen-jing Huang, Ying Li, Xi Wu

**Affiliations:** ^1^Acupuncture and Tuina School, The 3rd Teaching Hospital, Chengdu University of Traditional Chinese Medicine, No. 37 Shi'er Qiao Road, Chengdu, Sichuan 610075, China; ^2^Institute for Social Medicine, Epidemiology and Health Economics, Charité University Medical Center, 10117 Berlin, Germany

## Abstract

Chronic fatigue syndrome (CFS) is a debilitating and complex disorder characterized by profound fatigue with uncertain pathologic mechanism. Neuroimage may be an important key to unveil the central nervous system (CNS) mechanism in CFS. Although most of the studies found gray matter (GM) volumes reduced in some brain regions in CFS, there are many factors that could affect GM volumes in CFS, including chronic pain, stress, psychiatric disorder, physical activity, and insomnia, which may bias the results. In this paper, through reviewing recent literatures, we discussed these interferential factors, which overlap with the symptoms of CFS.

## 1. Background

Chronic fatigue syndrome (CFS) is an illness which can cause severe impairment in daily functioning, bringing heavy social and economic burden [1, 2]. In Japan, a total economic burden of CFS was estimated to be $4.0 billion per year [3]. The prevalence of CFS ranges from 0.007% to 2.8% in the general adult population [4–6], depending on the different definition [7], the varied target population, and the diverse study methods [8]. After all, most persons diagnosed as CFS are 30–40 years old, and most surveys demonstrate female preponderance [4, 6]. CFS also occurs in children and adolescents but at a lower rate [9].

CFS is a syndrome whose diagnosis is based on the clinical manifestation of the patients. There are a number of diagnostic criteria for CFS, and most of the researches apply the one given by the Centers for Disease Control and Prevention [10]. Owing to the causes of CFS that have not been identified, the treatment is also limited. The most popular therapeutical method is cognitive behavior treatment (CBT) [11]. However, a review of 14 studies of CFS reported that only 5% of patients fully recover after conventional treatment [12]. As an ancient traditional therapy, acupuncture got satisfied efficacy for CFS. Much research reported that acupuncture could relieve fatigue syndrome obviously, and the total effective rates are mostly over 85% [13, 14].

Meanwhile the pathological mechanism of CFS is still uncertain, major findings in etiology of recent studies include cerebral blood flow reduced in some brain regions [15–18], ventricular cerebrospinal fluid lactate increased [19], concentrations of corticosteroid declined combined with feedback of the hypothalamic-pituitary-adrenal (HPA) axis enhanced [20–22], brain-derived neurotrophic factor (BDNF) decreased [23, 24], serotonergic neurotransmitter system altered [25–27], and brain cytokines system activated [28, 29]. Although there are various findings, none of them can explain the mechanism and clinical symptoms in CFS comprehensively. In recent years, many studies on CFS focusing on the brain structures/systems benefit from rapid progress in neuroimaging techniques. The researchers wish that new findings could help to elucidate the central nervous system (CNS) pathologic mechanism of CFS.

Early findings of brain abnormalities in CFS were mostly focused on white matter hyperintensities (WMH). However, the inconsistent results [30–33] of WMH did not provide solid evidences to explain the pathologic changes in CNS of CFS. Then, it is a primary transfer to focus on the changes of gray matter (GM) volumes in CFS. We found 6 studies focusing on the change of GM volumes. Okada et al. [34] found in a voxel-based morphometric study of CFS that GM volumes reduced in bilateral prefrontal and that the affected areas extended from Brodmann area (BA) 8 to 9 in right cerebral hemisphere and from BA 9 to 11 in left. Puri et al. [35] also found that GM declined in occipital lobe, right angular gyrus, and posterior division of the left parahippocampal gyrus in a voxel-based morphometric 3-T MRI study. de Lange et al. [36] found that global GM volumes reduced by 8% compared to healthy controls and the rate of decline is 2.2 mL/year in a cohort study; moreover, the GM volumes could increase after cognitive behavioral therapy [37].

The occipital lobe, right angular gyrus, parahippocampal gyrus, and lateral prefrontal cortex in CFS showed abnormal activities in functional studies compared with healthy people [38, 39]. These changes consisted of the symptoms of cognition dysfunction, short memory loss, and so forth. The occipital lobe is an important part of visual processing in the brain. The right angular gyrus plays a critical role in perceptual sequence learning [40]. It also computes action awareness representations; in particular, it is involved in both awareness of discrepancy between intended and movement consequences and awareness of action authorship [41]. The parahippocampal gyrus is important in mnemonic functions such as encoding and retrieval. The lateral prefrontal cortex is an essential node of the network promoting executive functions [42]. Therefore, observing GM volumes provides an important method to interpret the CNS mechanism in CFS. However, not all the researches got positive results. Barnden et al. [43] reported their results: the total GM, WM, and cerebrospinal fluid (CSF) volumes from voxel-based morphometry (VBM) analysis showed no significant difference statistically and did not correlate with fatigue duration either.

Most of the results showed reductions of GM volumes, but the reductions were not involved in the same brain regions (Table 1). These inconsistent results made it difficult to draw a conclusion on CNS mechanism of CFS. This could be related to inconsistent design of studies such as sample size, age, proportion of genders, diagnosis criteria, CFS duration, and different accompanied symptoms. Although most researchers considered age as the confounding covariate when they did statistical analysis [36, 37] and some only recruited female patients or reconciled the proportion of the male versus the female between CFS and healthy controls so as to remove the influence of gender discrepancies [36, 37, 43], there are still some important factors related to the changes of GM volumes in CFS patients that are not taken into account. So in this paper, we review recent literatures and sum up the important aspects that are related to GM volumes and that coexist in CFS patients including chronic pain, personality, stress, psychiatric disorders, physical activity, and sleep (Table 2).

## 2. Chronic Pain

In CFS patients, 5 out of all 8 concomitant symptoms are pain related, including sore throat, tender glands, aching or stiff muscles, multijoint pain, and new headaches. According to the diagnosis criteria [10], CFS patient must have 4 concomitant symptoms, which means that the patients must have one pain symptom at least. However, current studies on CFS hardly have considered the role of pain. So further researches should elucidate the effect of chronic pain on pathologic progress of CFS, whether and how chronic pain changes the GM volumes in CFS patients.

Chronic pain is considered to be induced partly by maladaptive functional or structural plasticity of the nociceptive system that can occur at various sites from the spinal cord to the cerebral cortex [44]. MRI-based volumetric studies repeatedly found that patients with chronic pain show GM reductions in several brain areas belonging to the nociceptive system. Altered brain morphology has been described in several types of pain, such as chronic back pain [45–47], chronic tension-type headache [48], fibromyalgia [49, 50], migraine [51–53], and somatoform pain disorder [54]. The so-called “brain signature” of chronic pain has been suggested in cingulate cortex, prefrontal cortex, insula, and dorsal pons because different types of pain show GM alterations in these regions [55].

Different pain locations may have their specific brain regions. Some results suggest that GM reductions in the low back pain and headache groups are located on frontal regions, while GM reductions in the joint pain group may be more concentrated on parietal regions and the posterior cingulate cortex (PCC) [56]. Some evidences suggested that GM changes are neither preexisting nor due to irreversible cell damage [57, 58].

Whether direct correlation existed between GM volumes reduction and pain duration, frequency or intensity is still controversial. Previous studies reported positive correlations between GM volumes decrease and pain duration [59, 60], while the others did not [61].

Prefrontal cortex is the only region that changed in CFS overlapped with “brain signature.” Whether other regions in “brain signature” have changes in CFS and whether CFS pain has its specific brain regions are questions that need further studies to clear.

## 3. Personality

Harm avoidance (HA) is one of the three main dimensions of personality [62], which refers to an individual's tendency to inhibit behaviors and is expressed as an innate tendency to caution, apprehensiveness, and pessimism. It manifests as pessimistic worry in anticipation of future problems, passive avoidant behavior, fear of uncertainty, shyness of strangers, and rapid fatigability. HA is usually measured as an anxiety-related personality trait. CFS is also thought to be anxiety-related, in accordance with several features of HA personality trait, such as rapid fatigability. So we can presume that HA could contribute to most percentages of personality traits among CFS patients.

There is a hypothesis that individual differences in personality traits might be biologically determined and associated with volumetric variability in specific regions of the brain. Recently, several researches support this notion shown below.

A large sample of eighty-five young adult participants completed the Three-dimensional Personality Questionnaire (TPQ) in which their brains were imaged with MRI. A voxel-based correlation analysis was carried out between individuals' personality trait scores and GM volumes value extracted from 3D brain scans. HA showed a negative correlation with GM volumes in orbitofrontal, occipital, and parietal structures. And only in females there was a negative correlation between HA scores and GM volumes in the anterior prefrontal cortex [63].

Another study demonstrated that smaller right hippocampus was correlated with higher anxiety-related traits in both genders. Correlational analyses showed a significant negative correlation between the scores of HA and the regional GM volumes in left anterior prefrontal cortex in female but not in male subjects [64].

Interestingly, GM volumes in occipital and prefrontal cortex structures are also found decreasing in CFS patients. Because personalities are said to be heritable, stable across time, and dependent on genetic and neurobiological factors, whether CFS has the same characters is still unknown. In further studies, we should survey the HA prevalence rate in CFS patients and unveil the interaction between them.

## 4. Stress

The CFS patients are usually at the age of 30s to 40s, during which they endure more work and life stress [4, 6]. A case-control study suggested that stressful events and difficulties preceded the onset of CFS [65]. So stress is an important pathogenic factor of CFS. A longitudinal study on healthy postmenopausal women who had suffered from chronic stress over approximate 20 years reported decreased GM volumes in the right hippocampus which were consistent with previous human clinical MRI studies [66–68]. A secondary finding of the study was that chronic perceived stress predicted decreased GM volumes in the right orbitofrontal area of the prefrontal cortex.

## 5. Psychiatric Disorders

Anxiety is a major comorbidity in CFS [69] and a high proportion of patients with anxiety disorders complain of fatigue [70]. Indeed, patients with CFS have increased prevalence of temporary or lifetime mood disorders, primarily major depression, compared to other chronically ill subjects or healthy comparison subjects; 25% and 50%–75% of patients have a temporary or a lifetime history of major depression [71–73]. Generalized anxiety disorder and somatoform disorder also occur at a higher rate in chronic fatigue syndrome subjects than in the general population [69, 74]. In most [75, 76], but not all cases [77, 78], the mood or anxiety disorder precedes the onset of chronic fatigue syndrome. Although the major depression patients were excluded in most of the study, their anxiety and depression scores were still higher than those of healthy controls [43].

Recently, a large sample investigation including 640 subjects was administrated to measure the prevalence of specific cognition and behaviors in patients with CFS and to determine their association with comorbid anxiety or depression disorders. The results showed that 54% of the total sample had a diagnosis of CFS and no depression or anxiety disorder, 14% had CFS and one anxiety disorder, 14% had CFS and depressive disorder, and 18% had CFS and both depression and anxiety disorders [79].

A recent study also found that, compared to healthy controls, participants with major depression disease showed significant decreases of regional GM volumes in the amygdala, hippocampal, parahippocampal regions, ventral and medial temporal lobes, and insular cortex [80].

The changes in prefrontal cortex and parahippocampal are consistent with the known GM volumes reduction areas in CFS. We need to design a strict study to unveil whether CFS contributes to these changes either dependently or with coeffect with psychiatric disorders.

## 6. Physical Activity

Like chronic pain sufferers, CFS patients have been shown to avoid or fear physical activities [81]. van der Werf and his colleagues [82] conducted a long-term case-control study of daytime physical activity in 277 patients with CFS. The CFS patients in general were less active than healthy controls, with less intensity and shorter activity peaks that were in turn followed by longer rest periods.

The significantly positive correlations were found between local GM volumes and physical activity levels in parts of right prefrontal and cingulate cortex, as well as left prefrontal cortex, cingulate cortex, bilateral occipitotemporal regions, and cerebellum. The highest association was found in the right anterior frontal cortex. The present studies were partly overlapping with areas implicated in previous studies about the impact of physical activity on local GM volumes, including middle prefrontal gyrus, anterior cingulate cortex, and supplementary motor area [83, 84].

Healthy people take more physical activities than the patients of CFS, so they seem to have larger GM volumes naturally [34, 36]. Thus, the physical activity discrepancy should be considered when we design the neuroimaging research.

## 7. Insomnia

Unrefreshing sleep is the most prevalent of the 8 CFS case-defining symptoms, being endorsed by 87.5% of cases identified in population-based studies [6]. Reports showed that poor sleep or insomnia can also correlate with GM volumes. Altena et al. [85] found that insomnia patients had a smaller volume of GM than control subjects do in left orbitofrontal cortex (OFC), bilateral anterior precuneus of the parietal cortex, and bilateral posterior precuneus in the occipitoparietal cortex. No areas of higher GM volumes were found in the insomnia patients, compared with control participants [85]. One of the mechanisms in CFS is sleep architecture disorder [86]. So the effect of sleep on CFS should be researched deeply in neuroimage study.

## 8. Conclusion

Currently, acupuncture demonstrates more advantages than the other therapies, though this result should be further proved by more convincible evidences. Because of vague pathologic mechanism, it is urgent to develop robust diagnosis and evaluation methods. Neuroimage methods may provide a new angle to probe CFS. Although most studies found GM volumes reduced in some brain regions in CFS, as we discussed above, there are so many factors that could affect brain structure should have been considered. So far, no study has controlled all these factors. Thus, in further studies, we should improve research quality from the following points: (1) exclude factors which should be controlled, such as stress, psychiatric disorders, and physical activity; (2) study the effects of some important accompany symptoms on the occurrence and development of CFS, such as chronic pain and insomnia; (3) we need more large scale, perspective studies to prove our findings; (4) apply new imaging techniques and analyze methods, like diffusion tensor imaging, magnetic resonance spectroscopy, connectomics, and so forth to investigate CNS alterations of the disease and find specific pathological mechanism of CFS through which we could well understand and treat this disease in the future.

## Figures and Tables

**Table 1:** The gray matter volumes change in different researches.

Year	Title	Authors	Results			
			Gray matter	Regions	White matter	Regions
2004	Mechanisms underlying fatigue, a voxel-based morphometric study of chronic fatigue syndrome	Okada et al. [34]	Reduction	Bilateral prefrontal	No significance	

2005	Gray matter volume reduction in the chronic fatigue syndrome	de Lange et al. [36]	Reduction	Globe, no special regions	No significance	

2008	Increase in prefrontal cortical volume following cognitive behavioural therapy in patients with chronic fatigue syndrome	de Lange et al. [37]	Increased	Lateral prefrontal	No significance	

2011	A brain MRI study of chronic fatigue syndrome evidence of brainstem dysfunction and altered homeostasis	Barnden et al. [43]	No significance		No significance	

2012	Regional grey and white matter volumetric changes in myalgic encephalomyelitis (chronic fatigue syndrome), a voxel-based morphometry 3-T MRI study	Puri et al. [35]	Reduction	Occipital lobes, the right angular gyrus and the posterior division of the left parahippocampal gyrus	Reduction	Left occipital lobe

**Table 2:** The regions of gray matter volumes change with different factors.

Factors	Regions
Chronic pain	Cingulate cortex, prefrontal cortex, insula, and dorsal pons [55]

Personality	Orbitofrontal, occipital, and parietal structures; anterior prefrontal cortex (negatively related to women) [63]

Stress	Right orbitofrontal area of the prefrontal cortex [66–68]

Psychiatric disorder	Amygdala, hippocampal and parahippocampal, the ventral, medial temporal lobes, insular cortex [80]

Physical activity	Right prefrontal and cingulate cortex, left prefrontal cortex, cingulate cortex, bilateral occipitotemporal regions, and cerebellum, right anterior frontal cortex, middle prefrontal gyrus, anterior cingulate cortex, and supplementary motor area [83, 84]

Insomnia	Left orbitofrontal cortex, bilateral anterior precuneus of the parietal cortex, and bilateral posterior precuneus in the occipitoparietal cortex [85]

## Abbreviations

BA: Brodmann area
BDNF: Brain-derived neurotrophic factor
CFS: Chronic fatigue syndrome
CNS: Central nervous system
CSF: Cerebrospinal fluid
GM: Gray matter
HA: Harm avoidance
HPA: Hypothalamic-pituitary-adrenal
OFC: Orbitofrontal cortex
PCC: Posterior cingulate cortex
TPQ: Three-dimensional Personality Questionnaire
VBM: Voxel-based morphometry
WMH: White matter hyperintensities.
